# An innovative proposal for anophthalmic cavity reconstruction surgery with bone cement: a case report

**DOI:** 10.5935/0004-2749.20200006

**Published:** 2020

**Authors:** Letícia Aguiar Fonseca, Antônio Higor Marques Aragão, Álvaro Fernandes Ferreira, Bruno Fortaleza de Aquino Ferreira, Dácio Carvalho Costa

**Affiliations:** 1 Universidade Estadual do Ceará, Fortaleza, CE, Brazil; 2 Instituto Doutor José Frota, Fortaleza, CE, Brazil; 3 Hospital das Clínicas, São Paulo, SP, Brazil

**Keywords:** Eye evisceration, Orbital implants, Bone cements, Polymethyl methacrylate, Ophthalmologic surgical procedures, Case reports, Evisceração do olho, Implantes orbitários, Cimentos para ossos, Polimetil metacrilato, Procedimentos cirúrgicos oftalmológicos, Relatos de casos

## Abstract

We present a patient who underwent evisceration surgery after spontaneous rupture of the ocular globe due to long-data uncontrolled glaucoma, with posterior placement of an orbital implant made of a bone cement compound based on polymethylmethacrylate as alternative materials were not available. Such a compound is characterized by excellent biocompatibility and low cost, which makes it an interesting alternative for treatment. The anophthalmic socket was successfully filled, providing proper esthetic results and favorable conditions for the posterior scleral prosthesis implantation. No complications were observed during 10 months of follow-up. We believe that, in the absence of alternative materials, low-cost materials may be used in emergency settings to repair anophthalmic cavities and provide satisfactory functional and esthetic outcomes.

## Abstract (pt)

Apresentamos um paciente que foi submetido à cirurgia de evisceração após ruptura espontânea do globo ocular devido a glaucoma não controlado de longa data, com posterior colocação de implante orbital feito de cimento ósseo, composto à base de polimetilmetacrilato, diante da indisponibilidade de materiais alternativos. Tal composto se caracteriza pela excelente biocompatibilidade e baixo custo, o que o torna uma alternativa interessante para o tratamento. A cavidade anoftálmica foi preenchida com sucesso, fornecendo resultados estéticos adequados e condições favoráveis para o implante posterior de prótese escleral. Nenhuma complicação foi observada durante os 10 meses de seguimento. Acreditamos que, na ausência de materiais alternativos, materiais de baixo custo podem ser usados em situações emergenciais para preencher cavidades anoftálmicas e prover resultados funcionais e estéticos satisfatórios.

## INTRODUCTION

Evisceration is frequently necessary in emergency settings as a result of several conditions, such as trauma, glaucoma, neoplasia, endophthalmitis, and others^(1)^. After eye removal, it is necessary to replace the lost orbital volume with orbital implants as the execution of this procedure minimizes the risk of complications, such as contraction of the cavity with depression of the upper eyelid or retraction of the lower eyelid^(2)^.

Many different materials have been used to repair anophthalmic sockets, such as polymethylmethacrylate (PMMA), silicone, polyethylene, hydroxyapatite, glass, and others. Among all available implants, the ideal choice for orbital reconstruction currently is a matter of discussion^(3)^.

The high cost of integrated implants has resulted in the development of alternative materials capable of providing adequate functional and esthetic results at an affordable price for large-scale production in the context of public services^(4)^.

This report presents an innovative proposal for restoring anophthalmic cavities using an orbital implant made of a bone cement compound based on PMMA, which has the advantages of low-cost and excellent biocompatibility.

## CASE REPORT

A 65-year-old man presented to the ophthalmologic emergency department at Instituto Doutor José Frota (Fortaleza, Brazil) with spontaneous rupture of the eye secondary to long-term uncontrolled glaucoma. Evisceration was performed, followed by filling of the anophthalmic socket using bone cement (Osteo-Class^®^, Baumer, São Paulo, Brazil) due to lack of alternative implants in the institution.

**Figure 1:**
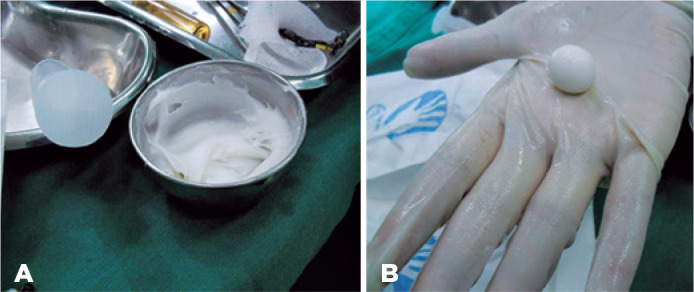
A) Mass formed after mixing the powder with the reagent. B) Appearance of the sphere after being cast.

This material is a compound based on PMMA and methylmethacrylate (MMA). Therefore, it is inert to the human eye. It is available as a powder (PMMA) with a liquid reagent (MMA) for preparation of the implant. After mixing the powder with the reagent, heat is released and the formed mass can be molded into the desired shape (in our case spheroid) and appropriate size, using a caliper to measure the ideal ball diameter for implant (Figure 1).

**Figure 2:**
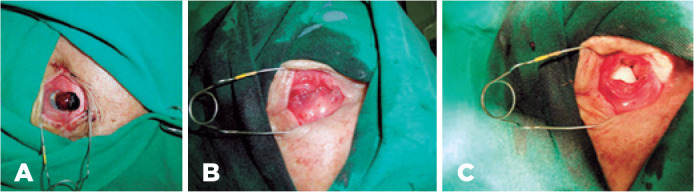
A) Appearance of the eyeball preoperatively. B) Anophthalmic cavity after evisceration. C) Implant appearance after being introduced into the cavity.

The anophthalmic socket was adequately filled with the orthopedic cement, providing proper esthetic results and suitable conditions for posterior scleral prosthesis implantation (Figure 2). The patient was reassessed in the immediate postoperative period and, progressed without complications, and was discharged 4 days postoperatively. No complications were observed at 10-month follow-up.

## DISCUSSION

For life-threatening diseases that affect intraorbital structures, such as advanced neoplasms or even irreparable damage from eye trauma or severe eye infections, emergent surgical removal of the eye is a painful but necessary decision that represents a challenge regarding repair^(3)^.

Despite the variety of materials currently available, the ideal choice remains debated as each type have their own strengths and weaknesses, and none to date has been able to combine all ideal characteristics, such as adequate biocompatibility, satisfactory volume filling, affordable costs, good mobility, and low complication rates^(5)^. Moreover, to our knowledge, no large series exists with long-term follow-up indicating that integrated implants perform better than nonintegrated implants.^(6)^.

Narikawa et al.^(2)^ analyzed the profile of patients with anophthalmia in a Brazilian region, demonstrating that PMMA was most commonly-used in surgeries. Affordable cost, ease of manufacture, and excellent biocompatibility with orbital tissues are some relevant characteristics of this material that makes its use very interesting in the context of developing countries. Further advantages include customized design, once it is possible to properly mold the material intraoperatively^(1)^. Complications associated with this material include dehiscence and spontaneous expulsion^(4)^. However, in our case, the implant remained stable and preserved the desired shape long term, and adequate cavity filling was maintained at a 10-month follow-up with no complications observed.

Despite the technological progress experienced over the last decades, the unavailability of materials at public hospitals is common, making it necessary to resort to alternative measures to attend patients who require urgent removal of the eye globe. In this case, owing to lack of orbital implants, we decided to use bone cement, a PMMA-based compound, to restore the anophthalmic socket and obtain a satisfactory cosmetic outcome and volume filling.

The success of previous experiences using materials containing PMMA reiterates its efficacy for public service use. Araf et al.^(7)^ presented a new model of a different orbital implant matching the reasonably priced acrylic implants (PMMA) with the advantages of porous polyethylene in a patient with previous implant extrusion. Excellent outcomes at a 6-month follow-up were achieved, demonstrating the possibility of using an economically accessible orbital implant with similar results compared to biointegrated implants. Furthermore, Jain and Jain^(8)^ described two patients who underwent orbital exenteration due to squamous cell carcinoma of the left upper eyelid with posterior acrylic resin prosthesis placement, and achieved satisfactory cosmetics results in both cases.

While to our knowledge no previous reports exist concerning the use of bone cement to restore anophthalmic sockets, this material has proven to be an interesting alternative in ophthalmology due to its successful applicability in other contexts. Vargas-Solalinde et al.^(9)^ described a well-succeeded procedure using a preconstructed bone cement implant to restore a blowout type orbital floor fracture.

PMMA use is approved by The Brazilian Health Surveillance Agency (ANVISA) for volumetric facial and body filling and its application for other types of reconstruction procedures is well established. The use of bone cement in orthopedic surgeries is well-known, being largely applied in cranioplasties and is considered as a first-line option in these operations when autogenous bone is not available. A long-term retrospective study showed mostly favorable results with bone cement in craniofacial surgery^(10)^.

Thus, we believe that low-cost alternative materials, such as bone cement, may become an alternative in emergency settings to individually restore anophthalmic sockets, allowing better rehabilitation, especially for low-income individuals who depend on public services. Further well-designed trials evolving a more significant number of patients are necessary to confirm the viability of this proposal.

## References

[1] Baino F, Perrero S, Ferraris S, Miola M, Balagna C, Verné E (2014). Biomaterials for orbital implants and ocular prostheses: overview
and future prospects. Acta Biomater.

[2] Narikawa S, Natsuaki KL, Fruet J, Padovani CR, Schellini SA. (2011). Perfil dos portadores de cavidade anoftálmica: estudo na
Faculdade de Medicina de Botucatu - UNESP. Arq Bras Oftalmol.

[3] Baino F, Protestio P. (2016). Orbital implants: State-of-the-art review with emphasis on
biomaterials and recent advances. Mater Sci Eng C Mater Biol Appl.

[4] Schellini SA, Hoyama E, Padovani CR, Ferreira VR, Roça R. (2000). Complicações com uso de esferas não integráveis e integráveis na
reconstrução da cavidade anoftálmica.. Arq Bras Oftalmol.

[5] Marano R, Tincani AJ. (2016). Is there an ideal implant for orbital reconstructions?
Prospective 64-case study. J Craniomaxillofac Surg.

[6] Schellini SA, El Dib R, Silva LR, Farat JG, Zhang Y, Jorge EC. (2016). Integrated versus non-integrated orbital implants for treating
anophthalmic sockets. Cochrane Database Syst Rev.

[7] Araf D, Assae OM, De Brito RV, Aquino-Júnior G, Da Silva TA. (2010). Implante orbital misto para reconstrução de cavidade anoftálmica:
relato de caso.. Arq Bras Oftalmol.

[8] Jain S, Jain P. (2016). Rehabilitation of orbital cavity after orbital exenteration using
polymethyl methacrylate orbital prosthesis. J Indian Prosthodont Soc.

[9] Vargas-Solalinde E, Huichapa-Padilla ME, Garza-Cantú D, Renya-Martinez VH, Alatorre-Ricardo J, González-Treviño JL. (2017). Bone cement implant as an alternative for orbital floor
reconstruction: a case report. Cir Cir.

[10] Marchac D, Greensmith A. (2008). Long-term experience with methylmethacrylate cranioplasty in
craniofacial surgery. J Plast Reconstr Aesthet Surg.

